# Distribution of Central Corneal Thickness and its Association with Ocular Parameters in a Large Central European Cohort: The Gutenberg Health Study

**DOI:** 10.1371/journal.pone.0066158

**Published:** 2013-08-01

**Authors:** Esther M. Hoffmann, Julia Lamparter, Alireza Mirshahi, Heike Elflein, René Hoehn, Christian Wolfram, Katrin Lorenz, Max Adler, Philipp S. Wild, Andreas Schulz, Barbara Mathes, Maria Blettner, Norbert Pfeiffer

**Affiliations:** 1 University Medical Centre, Department of Ophthalmology, Mainz, Germany; 2 University Medical Centre, Department of Internal Medicine II, Mainz, Germany; 3 University Medical Centre, Gutenberg Health Study, Study coordination/statistics, Department of Internal Medicine II, Mainz, Germany; 4 University Medical Centre, Department of Biostatistics and Epidemiology, Mainz, Germany; Justus-Liebig-University Giessen, Germany

## Abstract

**Main objective:**

To evaluate the distribution of central corneal thickness (CCT) in a large German cohort and to analyse its relationship with intraocular pressure and further ocular factors.

**Design:**

Population-based, prospective, cohort study.

**Methods:**

The Gutenberg Health Study (GHS) cohort included 4,698 eligible enrollees of 5,000 subjects (age range 35–74 years) who participated in the survey from 2007 to 2008. All participants underwent an ophthalmological examination including slitlamp biomicroscopy, intraocular pressure measurement, central corneal thickness measurement, fundus examination, and were given a questionnaire regarding glaucoma history. Furthermore, all subjects underwent fundus photography and visual field testing using frequency doubling perimetry.

**Results:**

Mean CCT was 557.3±34.3 µm (male) and 551.6±35.2 µm in female subjects (Mean CCT from right and left eyes). Younger male participants (35–44 years) presented slightly thicker CCT than those older. We noted a significant CCT difference of 4 µm between right and left eyes, but a high correlation between eyes (Wilcoxon test for related samples: p<0.0001). Univariable linear regression stratified by gender showed that IOP was correlated with CCT (p<0.0001). A 10 µm increase in CCT led to an increase in IOP between 0.35–0.38 mm Hg, depending on the eye and gender. Multivariable linear regression analysis revealed correlations between gender, spherical equivalent (right eyes), and CCT (p<.0001 and p = 0.03, respectively).

**Conclusions:**

We observed positive correlations between CCT and IOP and gender. CCT was not correlated with age, contact lens wear, positive family history for glaucoma, lens status, or iris colour.

## Introduction

Intraocular pressure readings are influenced by central corneal thickness (CCT) and may therefore affect diagnosis, screening, and the management of patients with glaucoma and ocular hypertension. There is evidence that IOP may be underestimated in patients with thinner and overestimated in patients with thicker corneas [1]. A good correlation between IOP measured by Goldmann applanation tonometry and intracamerally-measured IOP was demonstrated in patients with a normal corneal thickness of about 550 µm [2].

Central corneal thickness of ocular hypertensive patients is also presumed to be a powerful predictor of glaucoma development, as eyes with corneal thickness of 555 microns or less showed a greater risk of developing glaucoma than those with a corneal thickness of more than 588 microns [3].

Many factors can affect CCT in the general population, such as age, gender, environmental and genetic factors, and race. The Ocular Hypertension Treatment Study demonstrated a correlation between greater mean central corneal thickness and younger age, female gender, and diabetes. Patients with ocular hypertension had thicker corneas than the general population [4]. In contrast, other studies found a correlation between higher CCT and male gender and older age [5], [6].

Data on central corneal thickness have already been evaluated in several populations. Comparison among studies is difficult due to different measurement techniques (non-contact vs. contact measurements techniques) and missing weighting procedure in most population studies. Aghaian et al. investigated differences in central corneal thickness of 801 subjects, demonstrating that the CCT of Japanese participants was significantly lower than that of Caucasians, Chinese, Filipinos, and Hispanics, and greater than that of African Americans [7]. Compared to white subjects, African American subjects have thinner corneas [4].

There are few data available on the normal CCT in Indians. The Chennai Glaucoma Study is a population-based study of adults aged 40 years and older residing in the southern Indian state of Tamil Nadu. As part of a comprehensive eye examination, CCT and IOP measurements were obtained [5].

European studies that assessed CCT data are the European Glaucoma Prevention Study [8], [9], Reykjavik Eye Study [10] and Rotterdam study [11].

The purpose of this population-based, cross-sectional study is to evaluate the gender- and age-related distribution of central corneal thickness in a large German sample in the Rhine-Main region, and furthermore, to evaluate the relationship between central corneal thickness, intraocular pressure and other ocular factors.

## Materials and Methods

### The Gutenberg Health Study

The Gutenberg Health Study is a prospective, population-based cohort study being carried out in the Rhine-Main region of Germany (Rhineland-Palatinate). Its primary aim is to develop a new cardiovascular risk score, which takes into account classic, psychosocial, environmental and lifestyle risk factors, subclinical atherosclerotic disease, protein patterns and genetic variability concerning myocardial infarction and cardiovascular death as primary endpoints. Apoplexy, overall deaths, development of heart failure, and diabetes serve as secondary endpoints.

The population of interest is the population of Rhineland-Palatinate. 15,000 inhabitants in the districts Mainz and Mainz-Bingen aged between 35 and 74 years (equal distribution of subjects in each age group) have been selected as our study population. Two and a half years after baseline examination, participants will be contacted for a follow-up interview. Five years after baseline investigation, the study participants will be invited for a second follow-up, and the entire series of investigations will be repeated.

The structured series of investigations is performed within 5 hours.

Questionnaires on psychosocial work stress, physical activity, active and environmental smoking, nutrition, noise and air pollution will be assessed by a structured, computer-assisted, personal interview. Medical technical parameters include ankle-brachial index, anthropometric measurements, arterial waveform collection, blood pressure and heart rate measurements, sonography of the carotid arteries, echocardiography, electrocardiogram, endothelial function measurement, flow-mediated and nitro-mediated dilation, spirometry, expired carbon-monoxide measurement, laboratory parameters, biobanking and genotyping, as well as laboratory safety and routine parameters (electrolytes, kidney, liver, blood count coagulation, musculature, enzymes, inflammation, diabetes, lipids, homocysteine, thyroid, and oxidative stress).

### Ethics Statement

Written informed consent was obtained from each participant prior to any examination, according to the tenets of the Declaration of Helsinki. The study was approved by the Medical Ethics Committee of Mainz, Rhineland Palatinate, and by the local and federal data safety commissioners of the University Medical Center Mainz.

A sample of 5000 inhabitants within the Gutenberg Health Study (GHS) was included in this survey. 2540 male and 2460 female subjects between 35 and 75 years of age were examined between April 2007 and October 2008. Mean age was 56.0±10.9 (male) and 55.0±11.0 (female) years. Beside the extensive internal/general examination, all participants underwent an ophthalmological examination including visual acuity testing and refraction (Humphrey® Automated refractor/Keratometer (HARK) 599™, slitlamp biomicroscopy (Haag-Streit BM 900^®^, Bern Switzerland), intraocular pressure measurement (with a non-contact tonometer, NT 2000^TM^, Nidek Co./Japan), central corneal thickness and keratometry measurement (non-contact pachymetry with the Pachycam^TM^, Oculus, Wetzlar/Germany), and fundus examination. The measurement of corneal thickness is based on Scheimpflug images of a horizontal 4 mm cut through the corneal apex. With Scheimpflug acquisition, 600 absolute height values are analysed. The slitbeam illuminates the corneal surface to the corneal endothelium. The transparent cells of the cornea scatter the slit, leading to an apparently self-illuminated image of the cutting plane. This image is recorded by a camera angled at 45° whereas the image plane itself is located at 45° to the optical axis of the camera optics. This is required for defined cross-sectional cuts (Scheimpflug image). Participants were asked to fill out a questionnaire about their glaucoma history. Furthermore, all subjects underwent non-mydriatic fundus photography and visual field testing using frequency-doubling technology perimetry (Visucam *^ProNM^* ^,^ ™, and Humphrey Matrix Perimeter, Carl Zeiss Meditec AG, Jena, Germany). To measure intraocular pressure, we used the mean of three measurements within a range of 3 mm Hg for each eye, starting with the right eye. Iris colour was classified in blue, grey, green and brown by the ophthalmologist via an electronic case report file (eCRF). Spherical equivalent was calculated by adding the spherical correction value plus half the cylinder value.

### Inclusion and exclusion criteria

Subjects were randomly drawn from the local registry, Mainz, Germany and the Mainz-Bingen district. The sample was stratified 1∶1 for gender, residency, and age decades. Exclusion criteria were insufficient command of the German language to understand study documents, and computer-assisted interviews without translation, and the physical or psychological incapacity to travel to the study centre and/or to cooperate in the investigations. Participants with relevant corneal pathologies, corneal scarring, corneal dystrophies (such as cornea guttata/Fuchs endothelial dystrophy), and participants wearing hard contact lenses who had undergone laser in situ keratomileusis (LASIK), laser subepithelial keratomileusis (LASEK) or other refractive surgeries affecting the cornea were also excluded. Furthermore, participants with leucoma corneae, anophthalmia, or phthisis bulbi were excluded from the study. Measurements of central corneal thickness had to have a quality index of at least 90% as automatically calculated with each measurement. A quality index of 90% is recommended by the manufacturer's manual.

### Main Outcome Measures

Main outcome measures of this study are the association between central corneal thickness and ocular parameters such as refraction (spherical equivalent), intraocular pressure, contact lens wear, lens status, and iris colour. Furthermore, correlations between CCT and gender, age, reported history of glaucoma and/or existing antiglaucomatous medication are assessed.

### Statistical Analysis

All data underwent quality control by a central data management unit and were checked for completeness and correctness by predefined algorithms and quality plausibility controls. The sample size was defined by the sample size calculation of the primary endpoint (cardiovascular death). All data was weighted with the old European standard population. Descriptive statistics including means, standard deviation (SD), range and 95% reference range were performed to evaluate the distribution of central corneal thickness in the studied population. Data was stratified by age, gender and eye. Furthermore, linear regression analysis and multivariate regression analysis were performed to assess ophthalmological parameters possibly correlated with central corneal thickness measurements. Possible differences between right and left eyes were analysed using Wilcoxon tests for related samples. SAS (Version 9.2, SAS-Institute Inc., Cary, NC, USA) and SPSS (Version 15, SPSS Inc., Chicago, USA) statistical software packages were used for analysis.

## Results

Our population sample consisted of 2540 men and 2460 women. Mean age was 56.0±10.9 years (men) and 55.0±11.0 years (women), respectively. Men suffered more often from high blood pressure, diabetes, dyslipidemia, obesity, and showed a greater use of nicotine. Prevalence of eye variables such as glaucoma (defined by a history of glaucoma or by antiglaucomatous medication), contact lens wear, eye drop application, relevant corneal pathologies (status following excimer laser surgery, corneal clouding, corneal scars, and status following penetrating corneal injury), lens status (pseudophakia), and eye diseases in family history is presented in table 1. Glaucoma prevalence was higher in women than men (1.68% versus 1.34%) as defined by the patient or antiglaucomatous treatment. Women were contact lens wearers more often (6.51% versus 3.24%) and used eye drops more frequently than men (12.43% versus 6.79%). They also reported a family history of eye disease more frequently (12.63% versus 7.97%). More men had undergone cataract surgery than the women (1.76% versus 1.52%).

**Table 1:** Prevalence of selected eye parameters, stratified by gender and weighted with the old European standard population.

Weighted	All	Men	Women
Glaucoma	1.49%	1.34%	1.68%
Contact lenses	4.98%	3.24%	6.51%
Eye drops	9.68%	6.79%	12.43%
Relevant eye drops	1.72%	1.46%	2.03%
Corneal pathology	3.74%	4.59%	2.96%
Relevant corneal pathology	0.48%	0.48%	0.48%
Lens status (Pseudophakia)	1.63%	1.53%	1.76%
Family history of eye disease	10.45%	7.97%	12.63%
Relevant eye disease in family history	4.61%	3.79%	5.58%

Relative frequencies are shown.

Relevant eye drops: antiglaucomatous eye drops.

Eye drops in general: drops for dry-eye syndrome, general eye care products.

Relevant cornel pathologies: corneal scarring, corneal dystrophies (such as cornea guttata/Fuchs endothelial dystrophy).

Relevant eye disease in family history: history of glaucoma.

Measurements of central corneal thickness were available in 4758 right eyes and 4761 left eyes. Of these, 4708 right eyes and 4721 left eyes achieved the quality criteria (quality index of 90%) and were included in the analysis. Mean CCT was 554.2±34.9 µm in the total study sample (weighted for age and gender: 554.2±34.8 µm). Table 2 presents the weighted 5^th^ and 95^th^ percentile of CCT in men and women. Men had slightly thicker central corneal thickness (mean CCT: 500 and 613 µm, 5^th^ and 95^th^ percentile, respectively) than women (mean CCT: 496 and 610 µm, 5^th^ and 95^th^ percentile, respectively) in all age decades. By tendence, men between 35 and 44 years had thicker CCTs than older men (561.1 μm (493.6–628.8 μm) versus 554.1 μm (488.7–619.4 μm), 557.5 μm (488.7–626.3 μm), and 555.5 μm (488.8–622.2 μm)), respectively (see table 3).

**Table 2:** Distribution of central corneal thickness in the overall study sample in men and women.

Weighted	All	Men	Women
**CCTR**	495, 609 (5th, 95th percentile)	497, 610 (5th, 95th percentile)	493, 608 (5th, 95th percentile)
**CCTL**	498, 616 (5th, 95th percentile)	503, 618 (5th, 95th percentile)	496, 616 (5th, 95th percentile)
**CCTMean**	498, 612 (5th, 95th percentile)	500, 613 (5th, 95th percentile)	496, 610 (5th, 95th percentile)
**CCTMin**	494, 606 (5th, 95th percentile)	495, 608 (5th, 95th percentile)	492, 606 (5th, 95th percentile)
**CCTDelta**	1.00, 22.00 (5th, 95th percentile)	1.00, 23.00 (5th, 95th percentile)	1.00, 22.00 (5th, 95th percentile)

Values are weighted with the old European standard population between 35 and 74 years.

**Table 3:** Distribution of central corneal thickness in the sample population stratified by age and gender, after weighting procedure.

	Age decades				
	35–44	45–54	55–64	65–74	Total
CCTR (N = 4708)	95% reference range (Mean −1.96*SD, Mean +1.96*SD) is shown				
Men	558.7 (490.8–626.7)	552.3 (485.8–618.7)	555.6 (486.4–624.9)	553.7 (486.1–621.3)	555.2 (487.3–623.2)
Women	548.7 (481.1–616.4)	549.8 (479.7 (619.8)	549.2 (479. 9–618.4)	550.8 (478.8–622.8)	549.5 (480.0–618.9)
Total	**553 (484.1−621.6)**	**551.2 (483.3−619.2)**	**552.5 (482.9−622)**	**552.3 (482.4−622.3)**	**552.2 (483.3−621.1)**

CCTL (N = 4721)	95% reference range (Mean −1.96*SD, Mean +1.96*SD) is shown				
Men	563.3 (493.3–633.3)	556.0 (490.1–621.9)	559.5 (489.8–629.2)	557.2 (489.4 −625.1)	559.3 (490.7–627.9)
Women	553.6 (483.2–624.1)	553.6 (483.9–622.8)	553.0 (483.4–622.7)	555.2 (483.3–627.0)	553.6 (483.2–624.1)
Total	**557.5 (486.7−628.1)**	**550 (486.7−623.3)**	**556.4 (486.4−626.3)**	**556.3 (486.3−626.2)**	**556.2 (486.5−625.9)**

CCTMean (N = 4698)	95% reference range (Mean −1.96*SD, Mean +1.96*SD) is shown				
Men	561.1 (493.6–628.8)	554.1 (488.7–619.4)	557.5 (488.7–626.3)	555.5 (488.8–622.2)	557.3 (490–624.5)
Women	551.0 (483.8–618.2)	551.8 (481.9–621.7)	551.1 (482.7–619.5)	552.9 (481.7–624.2)	551.6 (482.6–620.5)
Total	**555.2 (486.8–623.5)**	**553.1 (485.8–620.4)**	**554.4 (485.5–623.3)**	**554.3 (485.2–623.4)**	**554.2 (485.9−622.5)**

CCTMin (N = 4698)	95% reference range (Mean −1.96*SD, Mean +1.96*SD) is shown				
Men	556.7 (489.1–624.4)	550.0 (484.1–615.8)	553.4 (485.5–621.3)	550.7 (484.5–617.0)	552.9 (485.8–620.1)
Women	546.8 (479.4–614.3)	547.6 (477.9–617.4)	546.6 (478.4–614.9)	548.5 (476.8–620.2)	547.3 (487.3–616.3)
Total	**550.9 (482.4–619.4)**	**549.0 (481.6–616.4)**	**550.1 (481.7–618.5)**	**549.7 (480.6–618.8.)**	**549.9 (481.7–618.2)**

CCT Δ (N = 4698)	*Median and Quartile intervals are shown (skewed distribution)*				
Men	8	7	7	8	7
	(3–12)	(3–11)	(3–12)	(3–14)	(3–12)
Women	7	7	7	7	7
	(3–11)	(3–12)	(3–12)	(4–12)	(3–12)
Total	7	7	7	7	7
	**(3–12)**	**(3–11)**	**(3–12)**	**(3–12)**	**(3–12)**

CCTR: Mean central corneal thickness of all right eyes.

CCTL: Mean central corneal thickness of all left eyes.

CCTMean: Mean central corneal thickness between right and left eyes (only analysed when both measurements were available).

CCTMin: The smaller central corneal thickness value between right and left eyes (only analysed when both measurements were available).

CCT **Δ**: Difference in micrometer between CCT of the right versus left eye (only analysed when both measurements were available).

Table 3 furthermore, presents the distribution of CCT in men and women stratified by age and gender including the 95% reference range. Smallest CCT of mean CCT ranged from 481.7 µm (at age 65–74) to 483.8 µm (at age 35–44), with women again having thinner CCTs than men. The range of thick mean CCT in the population was between 619 and 628 µm in men and between 618.2 and 624.2 µm in women. A difference in mean CCT was found between right and left eyes in the total study population. The difference in CCT between both eyes (CCT total) was 7 micrometers (median) with quartile intervals of 3 to 13 µm (skewed distribution), depending on the age decade. However, Wilcoxon test for related samples revealed a significant correlation between both eyes (p = 0.0001).

**Figure 1:**
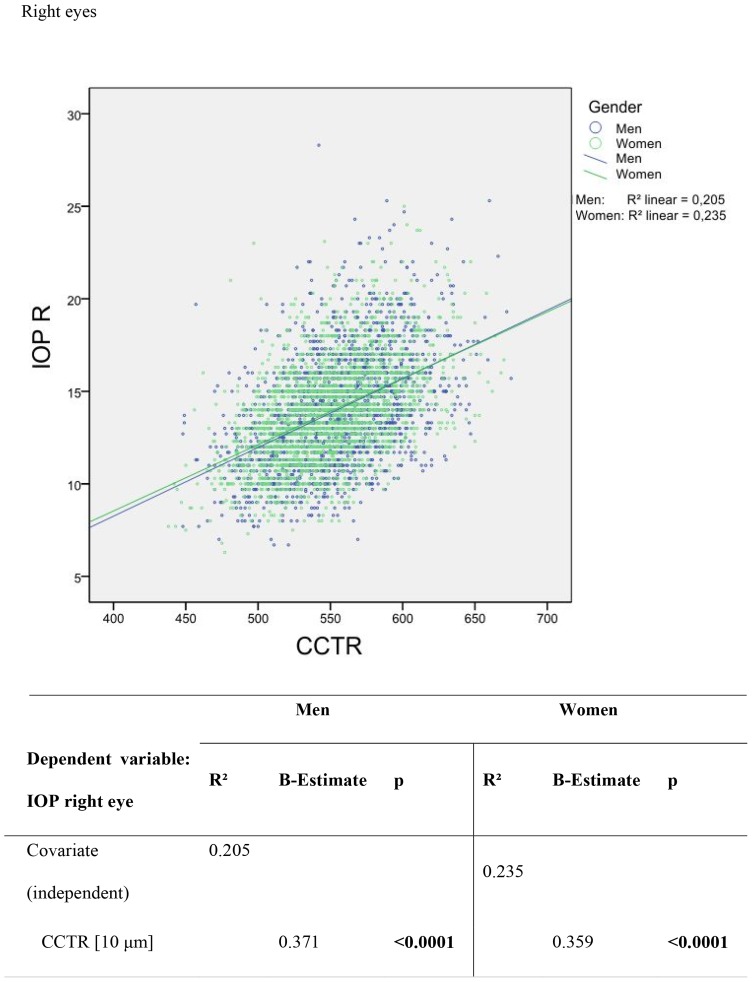
Scatter plots and univariable linear regression of the difference between non-contact applanation tonometry readings versus central corneal thickness in µm in right eyes.

**Figure 2:**
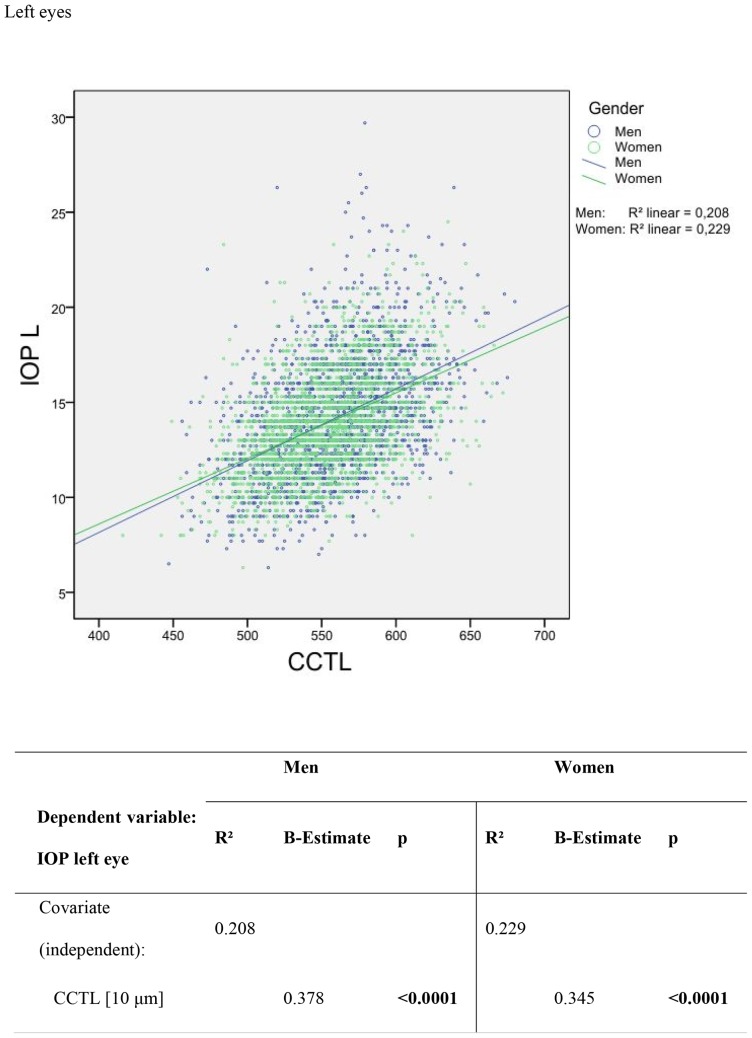
Scatter plots and univariable linear regression of the difference between non-contact applanation tonometry readings versus central corneal thickness in µm in left eyes.

Intraocular pressure was correlated with CCT. This relationship is shown in figures 1 and 2. In male right eyes, a 10 µm increase in CCT was correlated with an IOP increase of 0.37 mm Hg, r^2^ = 0.205 (female right eyes: 0.35 mm Hg change in IOP with a 10 µm CCT increase, r^2^ = 0.235). In the left eyes of all the men, a 10 µm increase in CCT correlated with an IOP increase of 0.38 mm Hg, r^2^ = 0.209 (women: 0.45 mm Hg change in IOP with a 10 µm CCT increase, r^2^ = 0.229). The correlation between different ophthalmological parameters and central corneal thickness was analysed by multivariate regression; results are in table 4a and 4b. Gender was correlated with central corneal thickness (regression coefficient −4.963 for right eyes and −4.735 for left eyes, p<0.0001), meaning that mean CCT in men was 4.963 μm (right eye) and 4.735 μm (left eye) thicker than in women.

**Table 4:** Results of multivariate regression analysis (adjusted for gender and age) evaluating the correlation between ophthalmological parameters and central corneal thickness measurements analysed in right and left eyes separately.

Right Eyes			
	R^2^	Reg.- coefficient	*p*-Value
**Parameter**	**0.0061**		
Gender		−4.963	**<0.0001**
Age in years		−0.060	0.2417
Spherical equivalent (right eye)		0.473	**0.0303**
Glaucoma*, no vs. yes		3.664	0.2571
Contact lenses, no vs. yes		0.100	0.9717
Lens status (right eye), phakic vs. pseudophakic		0.373	0.8960
Iris colour (right eye), light vs. dark		0.675	0.5476

Left Eyes			
	R^2^	Reg.- coefficient	*p*-Value
**Parameter**	**0.006**		
Gender		−4.735	**<0.0001**
Age in years		−0.070	0.1688
Spherical equivalent (right eye)		0.370	0.0885
Glaucoma*, no vs. yes		3.572	0.2752
Contact lenses, no vs. yes		−2.864	0.3089
Lens status (right eye), phakic vs. pseudophakic		2.146	0.4719
Iris colour (right eye), light vs. dark		1.066	0.3501

* Definition of glaucoma: as disclosed by the participant and/or by the use of antiglaucomatous medication.

Neither age, contact lens wear, positive family history for glaucoma, lens status, nor iris colour showed a correlation with CCT. For right eyes, refraction was positively correlated with CCT, as shown in table 4a (regression coefficient 0.473, p = 0.0303).

Glaucoma defined on the basis of disclosure by the participant and/or antiglaucomatous medication showed no correlation with CCT, as presented in table 5.

**Table 5:** Correlation between central corneal thickness and existence of glaucoma as disclosed by the participant and/or use of antiglaucomatous medication, stratified by gender.

Glaucoma			
	Glaucoma		
	No	Yes	*p*-Value
**CCTR** (µm)			
Men	555.3±34.6	551.9±36.5	0.51
Women	549.4±35.4	553.4±38.4	0.38
Total	**558.2±39.6**	**553.4±37.5**	**0.71**
**CCTL** (µm)			
Men	559.3±35.0	555.4±34.4	0.42
Women	553.5±35.9	558.2±39.6	0.32
Total	**556.2±35.5**	**557.6±37.3**	**0.68**

## Discussion

Central corneal thickness plays a major role in the diagnosis of glaucoma [3], [8], [9], [12]–[15]. It was the purpose of this study to evaluate central corneal thickness in a large population, since there are to date no population-based data on CCT in a German population. Mean CCT in the study population was 554.2±34.8 µm. That men have thicker CCT than women has been reported in other population-based studies as well [5], [8], [12]. One has to bear in mind that all comparisons among studies regarding mean CCT are dependent from the standard population used. We used the old European population. The mean CCT value in a German population is considerably higher than that in the Japanese Tajimi study (521±32 µm) [6], Central India Eye and Medical Study (514±33 µm) [12], Barbados Eye Study (530 µm, no standard deviation given) [16], Icelandic Reykjavik Eye Study (529±39 µm) [10], Chennai Glaucoma study (520.7±33.4 μm) [5] and in indigenous Australians (508±33 µm) [17]. Similar or slightly higher CCT values than in our study were found in the Beijing Eye Study (556.2±33.1 µm) [18], Rotterdam Eye Study (537 µm) [11], European Glaucoma Prevention Study (572.6±37.4 µm) [8], and the Ocular Hypertensive Treatment Study (578.1±36.8 µm) for those not developing glaucoma, 551.2±36.0 µm for those developing glaucoma [3], [13], and the Tehran Eye Study (555.6±39.9 µm) [19].

We noted an age dependency in mean CCT: 35-44-year-old participants had thicker CCT than participants aged 45–54. No further decrease in mean CCT was found in participants 45 years and older, and multivariate regression analysis revealed no correlation between CCT and age (Tables 5a and 5b). In contrast, a population-based study in 3280 Malayan subjects aged 40–80 years [20] revealed a continuous decrease in mean CCT over all age decades (40–49 years: 548.3 µm, 50–59 years: 544 µm, 60–69 years: 540.8 µm, 70–79 years: 533 µm). An age-related decrease in mean CCT has been shown in various clinical and population-based studies [12], [21], [22].

Previous population-based studies such as the Central India Eye and Medical Study [12] or Tehran Eye Study [19] found refraction not to be significantly correlated with central corneal thickness. The GHS Eye Survey found refraction to be positively correlated with CCT in right eyes in multivariate regression analysis after adjusting for gender and age.

Intraocular pressure was positively correlated with central corneal thickness. This was expected, since we used non-contact tonometry in this study, which is known to be positively correlated with CCT in the literature [23]–[25]. In general, there is good agreement between clinic-based and population-based studies reporting an increase in measured IOP as CCT increases. We observed results similar to those of Siganos [25] and Tonnu [24], whereas the Reykjavik Eye Study [10] reported a slightly flatter slope estimate compared to our results (women: 0.28 mm Hg an men: 0.22 mm Hg per 10 µm increase in CCT, respectively). There is evidence that non-contact tonometry is more susceptible than Goldmann applanation tonometry to CCT effects [24]. This may be due to the IOP measuring technique. In non-contact tonometry, the cornea is deformed over a shorter period (resulting in greater corneal stiffness) compared to Goldmann applanation tonometry, where measurement is relatively static [24], [26]. However, the study protocol required non-contact methods for the ophthalmological examinations to make them contrivable for ophthalmological technical assistants, too. New tonometry technologies have been developed recently, such as dynamic contour tonometry (DCT) and the ocular response analyzer (ORA), both being less dependent on CCT. The ORA (Reichert Inc., Depew, NY, USA), measures two applanation events, one as the pressure in the air jet rises and one as it falls. It thus measures corneal biomechanical properties such as corneal hysteresis and corneal elasticity. The reported effects of CCT on IOP measurements by DCT and ORA have been inconsistent among studies [27]–[31]. Some found IOP independent from CCT [25], [32]–[35], whereas a population-based cross-sectional study found DCT measurements slightly affected by CCT [36]. However, IOP measurements are affected by corneal thickness and other biomechanical properties, and CCT measurements will remain a key clinical factor when assessing an ophthalmological patient.

CCT measurements are important when monitoring glaucoma patients and ocular hypertensives, since studies have shown that thin CCT is associated with an increased glaucoma risk [3], [9], [37]. Furthermore, CCT measurements are helpful before and after kerato-refractive surgery and can assist in detecting endothelial disease and graft failure after penetrating keratoplasty [38].

Several instruments are available to measure corneal thickness with varying degrees of accuracy. Ultrasound pachymetry is commonly used to measure CCT (the “gold standard”) because it is easy to use and relatively inexpensive. Disadvantages of ultrasound pachymetry include the need to anaesthetise the cornea, cornea–probe contact, corneal indentation and the possible compression effect during measurement and corneal surface disturbance. There is also the risk of corneal epithelial damage and the transmission of infection. The reproducibility of ultrasound pachymetry measurements depends largely on examiner experience [38]. In our study, we used a non-contact pachymeter based on the Scheimpflug principle. Previous studies showed that Scheimpflug CCT measurements are more reproducible and repeatable than those obtained with US pachymetry, suggesting that the Scheimpflug camera is suitable for disease staging and follow-up. Furthermore, there is evidence that ultrasound pachymetry measurements are systematically thicker than Scheimpflug CCT measurements [38], [39]. In our study we used Scheimpflug imaging for CCT measurements, probably leading to less thicker CCTs than those obtained via ultrasound pachymetry techniques.

Based on a reported history of glaucoma and/or therapy with antiglaucomatous medication, prevalence for glaucoma in our cohort was 1.49% (Table 2). This is in line with results from other population-based studies such as the Beaver Dam Eye Study (overall prevalence of 2.1%, ranging from 0.9% in 43–54 year olds to 4.7% in people older than 75 years) [40], the Blue Mountains Eye Study (overall glaucoma prevalence in residents over 49 years of 3.0%, of whom 49% were diagnosed previously) [41], a meta-analysis of recent population-based studies, summarising prevalence estimates for glaucoma in the United States (overall prevalence of 1.89% in people 40 years and older) [42], the Egna-Neumarkt Study (overall prevalence of glaucoma: 1.4% in subjects older than 40 years) [43], in the Melbourne Visual Impairment Project (overall prevalence of 1.7% in the population, increasing steadily with age from 0.1% at ages 40 to 49 years to 9.7% in subjects aged 80 to 89 years) [44], and the Rotterdam Eye Study (overall prevalence of glaucoma of 0.8% ranging from 0.1% to 1.2% -depending on the definition for glaucoma in subjects older than 55 years) [45]. Higher prevalence of glaucoma has been detected in Japanese eyes [46], in Latinos of Mexican ancestry [47], and in populations of African origin [48]–[50] The varying results in prevalence data among the different population-based studies is related in part to the particular population, inclusion criteria, and the glaucoma definition. Although our cohort's prevalence data concurs with the glaucoma prevalence reported in the literature, one must bear in mind that – at least in this evaluation – glaucoma was not determined from optic nerve head data and/or visual field data. It however, had no impact on the results of this study. Data including glaucoma diagnosis by structure (optic nerve head) and function (FDT matrix) will be presented in future publications and are under preparation. Furthermore, results are strongly depending from the standard population used for comparison.

The strength of our study is that it is population-based and includes a high number of participants. Standardised and broad ophthalmological examination strengthens the validity of these results. The use of non-contact techniques is easy and feasible and of common use in population based studies. On the other side, this might be a limitation of this study, too. Although often used in (population-based) studies, non-contact tonometry has not been considered the goldstandard for IOP measurement. However, the protocol required a minimum of 3 measurements to decrease variability and the use of this technique had no impact on the purpose of the study.

In conclusion, the Gutenberg Health Study presents for the first time population-based CCT values in a representative German population of 5,000 middle-aged inhabitants. By weighting all data, results are reliable and applicable to the old European standard population. CCT was positively correlated with IOP and thus confirms results from other population-based surveys. Gender (men had thicker corneas) and spherical equivalent were correlated with CCT as well.

## References

[1] Whitacre MM, Stein RA, Hassanein K (1993) The effect of corneal thickness on applanation tonometry. Am J Ophthalmol 115: 592–596. 8488910 10.1016/s0002-9394(14)71455-2

[2] Boehm AG, Weber A, Pillunat LE, Koch R, Spoerl E (2008) Dynamic contour tonometry in comparison to intracameral IOP measurements. Invest Ophthalmol Vis Sci 49: 2472–2477. 18316699 10.1167/iovs.07-1366

[3] Gordon MO, Beiser JA, Brandt JD, Heuer DK, Higginbotham EJ, et al.. (2002) The Ocular Hypertension Treatment Study: baseline factors that predict the onset of primary open-angle glaucoma. Arch Ophthalmol 120: 714–720; discussion 829–730. 10.1001/archopht.120.6.714 12049575

[4] Brandt JD, Beiser JA, Kass MA, Gordon MO (2001) Central corneal thickness in the Ocular Hypertension Treatment Study (OHTS). Ophthalmology 108: 1779–1788. 11581049 10.1016/s0161-6420(01)00760-6

[5] Vijaya L, George R, Arvind H, Ve Ramesh S, Baskaran M, et al (2010) Central corneal thickness in adult South Indians: the Chennai Glaucoma Study. Ophthalmology 117: 700–704. 20079536 10.1016/j.ophtha.2009.09.025

[6] Tomidokoro A, Araie M, Iwase A (2007) Corneal thickness and relating factors in a population-based study in Japan: the Tajimi study. Am J Ophthalmol 144: 152–154. 17601447 10.1016/j.ajo.2007.02.031

[7] Aghaian E, Choe JE, Lin S, Stamper RL (2004) Central corneal thickness of Caucasians, Chinese, Hispanics, Filipinos, African Americans, and Japanese in a glaucoma clinic. Ophthalmology 111: 2211–2219. 15582076 10.1016/j.ophtha.2004.06.013

[8] Pfeiffer N, Torri V, Miglior S, Zeyen T, Adamsons I, et al (2007) Central corneal thickness in the European Glaucoma Prevention Study. Ophthalmology 114: 454–459. 17126403 10.1016/j.ophtha.2006.07.039

[9] Miglior S, Pfeiffer N, Torri V, Zeyen T, Cunha-Vaz J, et al (2007) Predictive factors for open-angle glaucoma among patients with ocular hypertension in the European Glaucoma Prevention Study. Ophthalmology 114: 3–9. 17070596 10.1016/j.ophtha.2006.05.075

[10] Eysteinsson T, Jonasson F, Sasaki H, Arnarsson A, Sverrisson T, et al (2002) Central corneal thickness, radius of the corneal curvature and intraocular pressure in normal subjects using non-contact techniques: Reykjavik Eye Study. Acta Ophthalmol Scand 80: 11–15. 11906297 10.1034/j.1600-0420.2002.800103.x

[11] Wolfs RC, Klaver CC, Vingerling JR, Grobbee DE, Hofman A, et al (1997) Distribution of central corneal thickness and its association with intraocular pressure: The Rotterdam Study. Am J Ophthalmol 123: 767–772. 9535620 10.1016/s0002-9394(14)71125-0

[12] Nangia V, Jonas JB, Sinha A, Matin A, Kulkarni M (2010) Central corneal thickness and its association with ocular and general parameters in Indians: the Central India Eye and Medical Study. Ophthalmology 117: 705–710. 20045561 10.1016/j.ophtha.2009.09.003

[13] Gordon MO, Torri V, Miglior S, Beiser JA, Floriani I, et al (2007) Validated prediction model for the development of primary open-angle glaucoma in individuals with ocular hypertension. Ophthalmology 114: 10–19. 17095090 10.1016/j.ophtha.2006.08.031 PMC1995665

[14] Miglior S, Torri V, Zeyen T, Pfeiffer N, Vaz JC, et al (2007) Intercurrent factors associated with the development of open-angle glaucoma in the European glaucoma prevention study. Am J Ophthalmol 144: 266–275. 17543874 10.1016/j.ajo.2007.04.040

[15] Leskea MC, Heijl A, Hyman L, Bengtsson B, Komaroff E (2004) Factors for progression and glaucoma treatment: the Early Manifest Glaucoma Trial. Curr Opin Ophthalmol 15: 102–106. 15021220 10.1097/00055735-200404000-00008

[16] Nemesure B, Wu SY, Hennis A, Leske MC (2003) Corneal thickness and intraocular pressure in the Barbados eye studies. Arch Ophthalmol 121: 240–244. 12583791 10.1001/archopht.121.2.240

[17] Landers JA, Billing KJ, Mills RA, Henderson TR, Craig JE (2007) Central corneal thickness of indigenous Australians within Central Australia. Am J Ophthalmol 143: 360–362. 17258537 10.1016/j.ajo.2006.09.047

[18] Zhang H, Xu L, Chen C, Jonas JB (2008) Central corneal thickness in adult Chinese. Association with ocular and general parameters. The Beijing Eye Study. Graefes Arch Clin Exp Ophthalmol 246: 587–592. 18196258 10.1007/s00417-007-0760-9

[19] Hashemi H, Yazdani K, Mehravaran S, KhabazKhoob M, Mohammad K, et al (2009) Corneal thickness in a population-based, cross-sectional study: the Tehran Eye Study. Cornea 28: 395–400. 19411957 10.1097/ICO.0b013e31818c4d62

[20] Wong TT, Wong TY, Foster PJ, Crowston JG, Fong CW, et al (2009) The relationship of intraocular pressure with age, systolic blood pressure, and central corneal thickness in an asian population. Invest Ophthalmol Vis Sci 50: 4097–4102. 19458324 10.1167/iovs.08-2822

[21] Kawase K, Tomidokoro A, Araie M, Iwase A, Yamamoto T (2008) Ocular and systemic factors related to intraocular pressure in Japanese adults: the Tajimi study. Br J Ophthalmol 92: 1175–1179. 18669541 10.1136/bjo.2007.128819

[22] Rochtchina E, Mitchell P, Wang JJ (2002) Relationship between age and intraocular pressure: the Blue Mountains Eye Study. Clin Experiment Ophthalmol 30: 173–175. 12010208 10.1046/j.1442-9071.2002.00519.x

[23] Kotecha A, White E, Schlottmann PG, Garway-Heath DF (2010) Intraocular pressure measurement precision with the Goldmann applanation, dynamic contour, and ocular response analyzer tonometers. Ophthalmology 117: 730–737. 20122737 10.1016/j.ophtha.2009.09.020

[24] Tonnu PA, Ho T, Newson T, El Sheikh A, Sharma K, et al (2005) The influence of central corneal thickness and age on intraocular pressure measured by pneumotonometry, non-contact tonometry, the Tono-Pen XL, and Goldmann applanation tonometry. Br J Ophthalmol 89: 851–854. 15965165 10.1136/bjo.2004.056622 PMC1772720

[25] Siganos DS, Papastergiou GI, Moedas C (2004) Assessment of the Pascal dynamic contour tonometer in monitoring intraocular pressure in unoperated eyes and eyes after LASIK. J Cataract Refract Surg 30: 746–751. 15093634 10.1016/j.jcrs.2003.12.033

[26] Hjortdal JO, Jensen PK (1995) In vitro measurement of corneal strain, thickness, and curvature using digital image processing. Acta Ophthalmol Scand 73: 5–11. 7627759 10.1111/j.1600-0420.1995.tb00004.x

[27] Realini T, Weinreb RN, Hobbs G (2009) Correlation of intraocular pressure measured with goldmann and dynamic contour tonometry in normal and glaucomatous eyes. J Glaucoma 18: 119–123. 19225347 10.1097/IJG.0b013e31817d23c7 PMC2704612

[28] Renier C, Zeyen T, Fieuws S, Vandenbroeck S, Stalmans I (2009) Comparison of ocular response analyzer, dynamic contour tonometer and Goldmann applanation tonometer. Int Ophthalmol. 10.1007/s10792-010-9377-9 20499265

[29] Bayer A, Sahin A, Hurmeric V, Ozge G (2010) Intraocular Pressure Values Obtained by Ocular Response Analyzer, Dynamic Contour Tonometry, and Goldmann Tonometry in Keratokonic Corneas. J Glaucoma. 10.1097/IJG.0b013e3181ca7aeb 20164793

[30] Leite MT, Alencar LM, Gore C, Weinreb RN, Sample PA, et al.. (2010) Comparison of Corneal Biomechanical Properties Between Healthy Blacks and Whites Using the Ocular Response Analyzer. Am J Ophthalmol 150: 163–168 e161. 10.1016/j.ajo.2010.02.024 PMC2912963 20538248

[31] Medeiros FA, Weinreb RN (2006) Evaluation of the influence of corneal biomechanical properties on intraocular pressure measurements using the ocular response analyzer. J Glaucoma 15: 364–370. 16988597 10.1097/01.ijg.0000212268.42606.97

[32] Kaufmann C, Bachmann LM, Thiel MA (2003) Intraocular pressure measurements using dynamic contour tonometry after laser in situ keratomileusis. Invest Ophthalmol Vis Sci 44: 3790–3794. 12939293 10.1167/iovs.02-0946

[33] Barleon L, Hoffmann EM, Berres M, Pfeiffer N, Grus FH (2006) Comparison of dynamic contour tonometry and goldmann applanation tonometry in glaucoma patients and healthy subjects. Am J Ophthalmol 142: 583–590. 17011849 10.1016/j.ajo.2006.05.030

[34] Pache M, Wilmsmeyer S, Lautebach S, Funk J (2005) Dynamic contour tonometry versus Goldmann applanation tonometry: a comparative study. Graefes Arch Clin Exp Ophthalmol 243: 763–767. 15756572 10.1007/s00417-005-1124-y

[35] Hager A, Loge K, Schroeder B, Fullhas MO, Wiegand W (2008) Effect of central corneal thickness and corneal hysteresis on tonometry as measured by dynamic contour tonometry, ocular response analyzer, and Goldmann tonometry in glaucomatous eyes. J Glaucoma 17: 361–365. 18703945 10.1097/IJG.0b013e31815c3ad3

[36] Francis BA, Hsieh A, Lai MY, Chopra V, Pena F, et al (2007) Effects of corneal thickness, corneal curvature, and intraocular pressure level on Goldmann applanation tonometry and dynamic contour tonometry. Ophthalmology 114: 20–26. 17070592 10.1016/j.ophtha.2006.06.047

[37] Miglior S, Zeyen T, Pfeiffer N, Cunha-Vaz J, Torri V, et al (2005) Results of the European Glaucoma Prevention Study. Ophthalmology 112: 366–375. 15745761 10.1016/j.ophtha.2004.11.030

[38] Grewal DS, Brar GS, Grewal SP (2010) Assessment of central corneal thickness in normal, keratoconus, and post-laser in situ keratomileusis eyes using Scheimpflug imaging, spectral domain optical coherence tomography, and ultrasound pachymetry. J Cataract Refract Surg 36: 954–964. 20494767 10.1016/j.jcrs.2009.12.033

[39] Barkana Y, Gerber Y, Elbaz U, Schwartz S, Ken-Dror G, et al (2005) Central corneal thickness measurement with the Pentacam Scheimpflug system, optical low-coherence reflectometry pachymeter, and ultrasound pachymetry. J Cataract Refract Surg 31: 1729–1735. 16246776 10.1016/j.jcrs.2005.03.058

[40] Klein BE, Klein R, Sponsel WE, Franke T, Cantor LB, et al (1992) Prevalence of glaucoma. The Beaver Dam Eye Study. Ophthalmology 99: 1499–1504. 1454314 10.1016/s0161-6420(92)31774-9

[41] Mitchell P, Smith W, Attebo K, Healey PR (1996) Prevalence of open-angle glaucoma in Australia. The Blue Mountains Eye Study. Ophthalmology 103: 1661–1669. 8874440 10.1016/s0161-6420(96)30449-1

[42] Friedman DS, Wolfs RC, O'Colmain BJ, Klein BE, Taylor HR, et al (2004) Prevalence of open-angle glaucoma among adults in the United States. Arch Ophthalmol 122: 532–538. 15078671 10.1001/archopht.122.4.532 PMC2798086

[43] Bonomi L, Marchini G, Marraffa M, Bernardi P, De Franco I, et al (1998) Prevalence of glaucoma and intraocular pressure distribution in a defined population. The Egna-Neumarkt Study. Ophthalmology 105: 209–215. 9479277 10.1016/s0161-6420(98)92665-3

[44] Wensor MD, McCarty CA, Stanislavsky YL, Livingston PM, Taylor HR (1998) The prevalence of glaucoma in the Melbourne Visual Impairment Project. Ophthalmology 105: 733–739. 9544649 10.1016/S0161-6420(98)94031-3

[45] Wolfs RC, Borger PH, Ramrattan RS, Klaver CC, Hulsman CA, et al (2000) Changing views on open-angle glaucoma: definitions and prevalences – The Rotterdam Study. Invest Ophthalmol Vis Sci 41: 3309–3321. 11006219

[46] Iwase A, Suzuki Y, Araie M, Yamamoto T, Abe H, et al (2004) The prevalence of primary open-angle glaucoma in Japanese: the Tajimi Study. Ophthalmology 111: 1641–1648. 15350316 10.1016/j.ophtha.2004.03.029

[47] Varma R, Ying-Lai M, Francis BA, Nguyen BB, Deneen J, et al (2004) Prevalence of open-angle glaucoma and ocular hypertension in Latinos: the Los Angeles Latino Eye Study. Ophthalmology 111: 1439–1448. 15288969 10.1016/j.ophtha.2004.01.025

[48] Tielsch JM, Katz J, Singh K, Quigley HA, Gottsch JD, et al (1991) A population-based evaluation of glaucoma screening: the Baltimore Eye Survey. Am J Epidemiol 134: 1102–1110. 1746520 10.1093/oxfordjournals.aje.a116013

[49] Hyman L, Wu SY, Connell AM, Schachat A, Nemesure B, et al (2001) Prevalence and causes of visual impairment in The Barbados Eye Study. Ophthalmology 108: 1751–1756. 11581045 10.1016/s0161-6420(01)00590-5

[50] Friedman DS, Jampel HD, Munoz B, West SK (2006) The prevalence of open-angle glaucoma among blacks and whites 73 years and older: the Salisbury Eye Evaluation Glaucoma Study. Arch Ophthalmol 124: 1625–1630. 17102012 10.1001/archopht.124.11.1625

